# Supplementary material for: An umbrella review of the evidence associating diet and cancer risk at 11 anatomical sites
Source: Nat Commun. 2021 Jul 28;12:4579. doi: 10.1038/s41467-021-24861-8 (PMC8319326; doi:10.1038/s41467-021-24861-8)
Supplement: Supplementary file 1 — Supplementary Information [file 41467_2021_24861_MOESM1_ESM.pdf]

Supplementary Methods

The current umbrella review used data from the WCRF Third Expert Report. The review protocols of this report are available online at https://www.wcrf.org/dietandcancer/resources-and-toolkit

The literature search was conducted in PubMed using a tested search strategy that contains medical subject headings (MeSH terms) and text words for a broad range of dietary factors and cancers with an end date pertinent to the review (see search terms below). Reference lists from published reviews, meta-analyses, and pooled projects were screened for additional relevant publications. There was no language restriction to the search. Authors were not contacted for further information.

Inclusion criteria of studies were: 1) Randomized controlled trials, cohort studies, case-cohort studies, nested case-control studies, or studies that pooled individual data from studies of these designs, in humans; 2) Studies that investigated the associations between dietary factors and cancer risk; and 3) Studies that reported relative risk (RR), hazard ratio (HR), or odds ratio (OR) as measures of association, with its 95% confidence interval or P-value.

Exclusion criteria were: 1) Ecologic, cross-sectional, and case-control studies; 2) articles published as comments, reviews, news or conference abstracts. In the case of multiple publications from the same study or overlapping populations between the publications, the publication with more number of cases was selected.

For a study to be included in a dose-response meta-analysis, either a dose-response slope or information including relative risk estimates, number of cases, non-cases, or person years-at risk, and intake values for at least three exposure categories are required. If the information is not provided in the study or could not be estimated using standard procedures, the study is excluded from the meta-analysis.

Search terms for the dietary factors:
#1 diet therapy[MeSH Terms] OR nutrition[MeSH Terms]

#2 diet[tiab] OR diets[tiab] OR dietetic[tiab] OR dietary[tiab] OR eating[tiab] OR intake[tiab] OR nutrient*[tiab] OR nutrition[tiab] OR vegetarian*[tiab] OR vegan*[tiab] OR "seventh day adventist"[tiab] OR macrobiotic[tiab]

#3 “food and beverages” [MeSH Terms]

#4 food*[tiab] OR cereal*[tiab] OR grain*[tiab] OR granary[tiab] OR wholegrain[tiab] OR wholewheat[tiab] OR roots[tiab] OR plantain*[tiab] OR tuber[tiab] OR tubers[tiab] OR vegetable*[tiab] OR fruit*[tiab] OR pulses[tiab] OR beans[tiab] OR lentils[tiab] OR chickpeas[tiab] OR legume*[tiab] OR soy[tiab] OR soya[tiab] OR nut[tiab] OR nuts[tiab] OR peanut*[tiab] OR groundnut*[tiab] OR (seeds[tiab] AND (diet*[tiab] OR food*[tiab])) OR meat[tiab] OR beef[tiab] OR pork[tiab] OR lamb[tiab] OR poultry[tiab] OR chicken[tiab] OR turkey[tiab] OR duck[tiab] OR (fish[tiab] AND (diet*[tiab] OR food*[tiab])) OR ((fat[tiab] OR fats[tiab] OR fatty[tiab]) AND (diet*[tiab] OR food*[tiab] OR adipose[tiab] OR blood[tiab] OR serum[tiab] OR plasma[tiab])) OR egg[tiab] OR eggs[tiab] OR bread[tiab] OR (oils[tiab] AND (diet*[tiab] OR food*[tiab] OR adipose[tiab] OR blood[tiab] OR serum[tiab] OR plasma[tiab])) OR shellfish[tiab] OR seafood[tiab] OR sugar[tiab] OR

syrup[tiab] OR dairy[tiab] OR milk[tiab] OR herbs[tiab] OR spices[tiab] OR chilli[tiab] OR chillis[tiab] OR pepper*[tiab] OR condiments[tiab] OR tomato*[tiab]

#5 fluid intake[tiab] OR water[tiab] OR drinks[tiab] OR drinking[tiab] OR tea[tiab] OR coffee[tiab] OR caffeine[tiab] OR juice[tiab] OR beer[tiab] OR spirits[tiab] OR liquor[tiab] OR wine[tiab] OR alcohol[tiab] OR alcoholic[tiab] OR beverage*[tiab] OR (ethanol[tiab] AND (drink*[tiab] OR intake[tiab] OR consumption[tiab])) OR yerba mate[tiab] OR ilex paraguariensis[tiab]

#6 food preservation[MeSH Terms]

#7 (mycotoxin*[tiab] OR aflatoxin*[tiab] OR pickled[tiab] OR bottled[tiab] OR bottling[tiab] OR canned[tiab] OR canning[tiab] OR vacuum pack*[tiab] OR refrigerate*[tiab] OR refrigeration[tiab] OR cured[tiab] OR smoked[tiab] OR preserved[tiab] OR preservatives[tiab] OR nitrosamine[tiab] OR hydrogenation[tiab] OR fortified[tiab] OR additive*[tiab] OR colouring*[tiab] OR coloring*[tiab] OR flavouring*[tiab] OR flavoring*[tiab] OR nitrates[tiab] OR nitrites[tiab] OR solvent[tiab] OR solvents[tiab] OR ferment*[tiab] OR processed[tiab] OR antioxidant*[tiab] OR genetic modif*[tiab] OR genetically modif*[tiab] OR vinyl chloride[tiab] OR packaging[tiab] OR labelling[tiab] OR phthalates[tiab]) AND (diet*[tiab] OR food*[tiab] OR adipose[tiab] OR blood[tiab] OR serum[tiab] OR plasma[tiab])

#8 cookery[MeSH Terms]

#9 cooking[tiab] OR cooked[tiab] OR grill[tiab] OR grilled[tiab] OR fried[tiab] OR fry[tiab] OR roast[tiab] OR bake[tiab] OR baked[tiab] OR stewing[tiab] OR stewed[tiab] OR casserol*[tiab] OR broil[tiab] OR broiled[tiab] OR boiled[tiab] OR ((microwave[tiab] OR microwaved[tiab] OR re-heating[tiab] OR reheating[tiab] OR heating[tiab] OR re-heated[tiab] OR heated[tiab]) AND (diet*[tiab] OR food*[tiab])) OR poach[tiab] OR poached[tiab] OR steamed[tiab] OR barbecue*[tiab] OR chargrill*[tiab] OR heterocyclic amines[tiab] OR polycyclic aromatic hydrocarbons[tiab]

#10 ((carbohydrates[MeSH Terms] OR proteins[MeSH Terms]) AND (diet*[tiab] OR food*[tiab])) OR sweetening agents[MeSH Terms]

#11 (salt[tiab] OR salting[tiab] OR salted[tiab] OR fiber[tiab] OR fibre[tiab] OR polysaccharide*[tiab] OR starch[tiab] OR starchy[tiab] OR carbohydrate*[tiab] OR lipid*[tiab] OR linoleic acid*[tiab] OR sterols[tiab] OR stanols[tiab] OR sugar*[tiab] OR sweetener*[tiab] OR saccharin*[tiab] OR aspartame[tiab] OR acesulfame[tiab] OR cyclamates[tiab] OR maltose[tiab] OR mannitol[tiab] OR sorbitol[tiab] OR sucrose[tiab] OR xylitol[tiab] OR cholesterol[tiab] OR protein[tiab] OR proteins[tiab] OR hydrogenated dietary oils[tiab] OR hydrogenated lard[tiab] OR hydrogenated oils[tiab]) AND (diet*[tiab] OR food*[tiab] OR adipose[tiab] OR blood[tiab] OR serum[tiab] OR plasma[tiab])

#12 vitamins[MeSH Terms]

#13 supplements[tiab] OR supplement[tiab] OR vitamin*[tiab] OR retinol[tiab] OR carotenoid*[tiab] OR tocopherol[tiab] OR folate*[tiab] OR folic acid[tiab] OR methionine[tiab] OR riboflavin[tiab] OR thiamine[tiab] OR niacin[tiab] OR pyridoxine[tiab] OR cobalamin[tiab] OR mineral*[tiab] OR (sodium[tiab] AND (diet*[tiab] OR food*[tiab]))

OR iron[tiab] OR ((calcium[tiab] AND (diet*[tiab] OR food*[tiab] OR supplement*[tiab])) OR selenium[tiab] OR (iodine[tiab] AND (diet*[tiab] OR food*[tiab] OR supplement*[tiab] OR deficiency)) OR magnesium[tiab] OR potassium[tiab] OR zinc[tiab] OR copper[tiab] OR phosphorus[tiab] OR manganese[tiab] OR chromium[tiab] OR phytochemical[tiab] OR allium[tiab] OR isothiocyanate*[tiab] OR glucosinolate*[tiab] OR indoles[tiab] OR polyphenol*[tiab] OR phytestrogen*[tiab] OR genistein[tiab] OR saponin*[tiab] OR coumarin*[tiab] OR lycopene[tiab]

Search terms, end date, and number of included publications in the mouth, pharyngeal, and laryngeal cancer systematic literature review:
#1 Laryngeal Neoplasms[MeSH] OR Pharyngeal Neoplasms[MeSH] OR Mouth Neoplasms[MeSH] OR Tongue Neoplasms[MeSH] OR Oropharyngeal Neoplasms[MeSH] OR Hypopharyngeal Neoplasms[MeSH]

#2 malign*[tiab] OR cancer*[tiab] OR carcinoma*[tiab] OR tumor*[tiab] OR tumour*[tiab] OR adenocarcinoma*[tiab] OR carcinoma, squamous cell*[tiab] OR carcinoma, small cell*[tiab]

#3 larynx[tiab] OR pharynx[tiab] OR laryngeal[tiab] OR pharyngeal[tiab] OR hypopharyngeal[tiab] OR oropharyngeal[tiab] OR mouth[tiab] OR tongue[tiab]

#4 #2 AND #3

#5 Oral Leukoplakia[MeSH]

#6 oral cancer*[tiab] OR oral carcinoma*[tiab] OR oral leukoplakia*[tiab]

#7 #1 OR #4 OR #5 OR #6

Searched up to 30 April 2015, 56 publications were included in the systematic literature review.

Search terms, end date, and number of included publications in the esophageal cancer systematic literature review:
#1 Esophageal Neoplasms [MeSH]

#2 Esophag*[tiab] OR oesophag*[tiab] OR upper aero digestive tract[tiab]

#3 malign*[tiab] OR cancer*[tiab] OR carcinoma*[tiab] OR tumor*[tiab] OR tumour*[tiab] OR adenocarcinoma*[tiab] OR carcinoma, squamous cell*[tiab] OR carcinoma, small cell*[tiab] OR high grade dysplasia[tiab]

#4 #2 AND #3

#5 Esophagogastric neoplasm*[tiab] OR esophagogastric cancer*[tiab] OR esophagogastric carcino* OR esophagogastric tumo*[tiab] OR esophagogastric metasta* [tiab] OR esophagogastric malign*[tiab] OR esophagogastric adenocarcinoma* [tiab] OR esophagogastric neoplasm*[tiab]

#6 Esophago gastric cancer*[tiab] OR esophago gastric carcino* OR esophago gastric tumo*[tiab] OR esophago gastric metasta* [tiab] OR esophago gastric malign*[tiab] OR esophago gastric adenocarcinoma* [tiab] OR Barrett's adenocarcinoma [tiab]

#7 Oesophagogastric neoplasm*[tiab] OR oesophagogastric cancer*[tiab] OR oesophagogastric carcino* OR oesophagogastric tumo*[tiab] OR oesophagogastric metasta* [tiab] OR oesophagogastric malign*[tiab] OR oesophagogastric adenocarcinoma* [tiab]

#8 Oesophago gastric neoplasm*[tiab] OR oesophago gastric cancer*[tiab] OR oesophagogastric carcino* OR oesophago gastric tumo*[tiab] OR oesophagogastric metasta* [tiab] OR oesophago gastric malign*[tiab] OR oesophagogastric adenocarcinoma* [tiab]

#9 #5 OR #6 OR #7 OR #8

#10 #1 OR #4 OR #9

Searched up to 28 February 2014, 97 publications were included in the systematic literature review.

Search terms, end date, and number of included publications in the stomach cancer systematic literature review:

#1 Stomach neoplasms[MeSH Terms]

#2 Stomach neoplasm*[tiab] OR stomach cancer*[tiab] OR stomach carcino* OR stomach tumo*[tiab] OR stomach metasta* [tiab] OR stomach malign*[tiab] OR stomach adenocarcinoma* [tiab]

#3 Gastric neoplasm* [tiab] OR gastric cancer*[tiab] OR gastric carcino* [tiab] or gastric tumo*[tiab] OR gastric metasta*[tiab] OR gastric malign*[tiab] OR gastric adenocarcinoma* [tiab]

#4 Gastrointestinal neoplasms[mesh terms] OR gastrointestinal neoplas*[tiab] OR gastrointestinal cancer*[tiab] OR gastrointestinal carcino*[tiab] OR gastrointestinal tumo*[tiab] OR gastrointestinal metasta*[tiab] OR gastrointestinal malign*[tiab] OR gastrointestinal adenocarcinoma*[tiab]

#5 Digestive tract neoplasm*[tiab] OR digestive tract cancer*[tiab] OR digestive tract carcino*[tiab] OR digestive tract tumo*[tiab] OR digestive tract metasta*[tiab] OR digestive tract malign*[tiab] OR digestive tract adenocarcinoma*[tiab]

#6 Alimentary tract neoplasm*[tiab] OR alimentary tract cancer*[tiab] OR alimentary tract carcino*[tiab] OR alimentary tract tumo*[tiab] OR alimentary tract metasta*[tiab] OR alimentary tract malign* OR alimentary tract adenocarcinoma*[tiab]

#7 Esophagogastric neoplasm*[tiab] OR esophagogastric cancer*[tiab] OR esophagogastric carcino* OR esophagogastric tumo*[tiab] OR esophagogastric metasta* [tiab] OR esophagogastric malign*[tiab] OR esophagogastric adenocarcinoma* [tiab] OR esophagogastric neoplasm*[tiab]

#8 Esophago gastric cancer*[tiab] OR esophago gastric carcino* OR esophago gastric tumo*[tiab] OR esophago gastric metasta* [tiab] OR esophago gastric malign*[tiab] OR esophago gastric adenocarcinoma* [tiab]

#9 Oesophagogastric neoplasm*[tiab] OR oesophagogastric cancer*[tiab] OR oesophagogastric carcino* OR oesophagogastric tumo*[tiab] OR oesophagogastric metasta* [tiab] OR oesophagogastric malign*[tiab] OR oesophagogastric adenocarcinoma* [tiab]

#10 Oesophago gastric neoplasm*[tiab] OR oesophago gastric cancer*[tiab] OR oesophago gastric carcino* OR oesophago gastric tumo*[tiab] OR oesophago gastric metasta* [tiab] OR oesophago gastric malign*[tiab] OR oesophago gastric adenocarcinoma* [tiab]

#11 Stomach adenoma*[tiab] OR gastric adenoma*[tiab] OR gastrointestinal adenoma*[tiab] OR digestive tract adenoma*[tiab] OR alimentary tract adenoma*[tiab] OR esophagogastric adenoma*[tiab] OR esophagogastric adenoma*[tiab] OR oesophagogastric adenoma*[tiab] OR oesophagogastric adenoma*[tiab]

#12 #1 OR #2 OR #3 OR #4 OR #5 OR #6 OR #7 OR #8 OR #9 OR #10 OR #11

Searched up to 28 February 2014, 250 publications were included in the systematic literature review.

Search terms, end date, and number of included publications in the colorectal cancer systematic literature review:
#1 Colorectal neoplasms [MeSH] OR intestinal polyps [MeSH]

#2 malign* [tiab] OR neoplasm* [tiab] OR carcinoma* [tiab] OR cancer* [tiab] OR tumor* [tiab] OR tumour* [tiab] OR polyp* [tiab]

#3 colon [tiab] OR rectum [tiab] OR rectal [tiab] OR colorectum [tiab] OR colorectal [tiab] OR large bowel [tiab] OR large intestine [tiab] OR gut [tiab]

#4 #1 OR (#2 AND #3)

Searched up to 30 April 2015, 275 publications were included in the systematic literature review.

Search terms, end date, and number of included publications in the liver cancer systematic literature review:
#1 liver neoplasms [MeSH]

#2 malign*[tiab] OR neoplasm*[tiab] OR carcinoma*[tiab] OR cancer*[tiab] OR tumor*[tiab] OR tumour*[tiab] OR angiosarcoma[tiab]

#3 liver[tiab] OR hepatocellular[tiab] OR cholangio*[tiab] OR hepatoblastoma[tiab] OR hepatic[tiab] OR hepatoma[tiab] OR hepatocarcinoma[tiab]

#4 #1 OR (#2 AND #3)

Searched up to 31 March 2013, 105 publications were included in the systematic literature review.

Search terms, end date, and number of included publications in the gallbladder cancer systematic literature review:
#1 Gallbladder neoplasm [MeSH]

#2 malign*[tiab] OR neoplasm*[tiab] OR carcinoma*[tiab] OR cancer*[tiab] OR tumor*[tiab] OR tumour*[tiab]

#3 gallbladder[tiab] OR biliary tract[tiab] OR bile ducts[tiab]

#4 #1 OR (#2 AND #3)

Searched up to 31 March 2013, 37 publications were included in the systematic literature review.

Search terms, end date, and number of included publications in the lung cancer systematic literature review:
#1 lung neoplasm [MeSH Terms] OR (lung AND (carcinoma[tiab] OR neoplasm*[tiab] OR tumor*[tiab]))

Searched up to 30 September 2014, 107 publications were included in the systematic literature review.

Search terms, end date, and number of included publications in the skin cancer systematic literature review:
#1 neoplasm[tiab] OR Skin cancer[tiab]

#2 skin[tiab] AND ("Basal cell carcinoma"[tiab] OR "squamous cell carcinoma"[tiab])

#3 Skin[tiab] AND (tumour*[tiab] OR tumor*[tiab])

#4 Skin[tiab] AND ("basal cell epithelioma"[tiab] OR "squamous cell epithelioma"[tiab])

#5 #1 OR #2 OR #3 OR #4

Searched up to 19 April 2016, 173 publications were included in the systematic literature review.

Search terms, end date, and number of included publications in the breast cancer systematic literature review:
#1 Breast Neoplasms [MeSH Terms]

#2 Breast AND (cancer* OR neoplasm* OR tumour* OR tumor* OR carcinoma* OR adenocarcinoma*)

#3 mammary AND (cancer* OR neoplasm* OR tumour* OR tumor* OR carcinoma* OR adenocarcinoma*)

#4 #1 OR #2 OR #3

Searched up to 30 April 2015, 900 publications were included in the systematic literature review.

Search terms, end date, and number of included publications in the kidney cancer systematic literature review:

#1 renal neoplasm [MeSH terms] OR kidney neoplasm [MeSH terms] OR urethral neoplasm [MeSH terms]

#2 kidney OR renal OR urethral AND (malign* [tiab] OR cancer*[tiab] OR carcinoma*[tiab] OR tumor*[tiab] OR tumour*[tiab] OR adenocarcinoma* OR sarcoma)

#3 hypernephroma* OR grawitz tumo* OR wilms tumo* OR nephroma*

#4 #1 OR #2 OR #3

Searched up to 31 March 2013, 74 publications were included in the systematic literature review.

Search terms, end date, and number of included publications in the urinary bladder cancer systematic literature review:

#1 urinary bladder neoplasm [MeSH]

#2 malign* [tiab] OR cancer*[tiab] OR carcinoma*[tiab] OR tumor*[tiab] OR tumour*[tiab]

#3 bladder [tiab] OR urinary [tiab] OR urothelial*[tiab] OR urologic*[tiab] OR urethral [tiab]

#4 #2 AND #3

#5 #1 OR #4

Searched up to 31 July 2013, 33 publications were included in the systematic literature review.

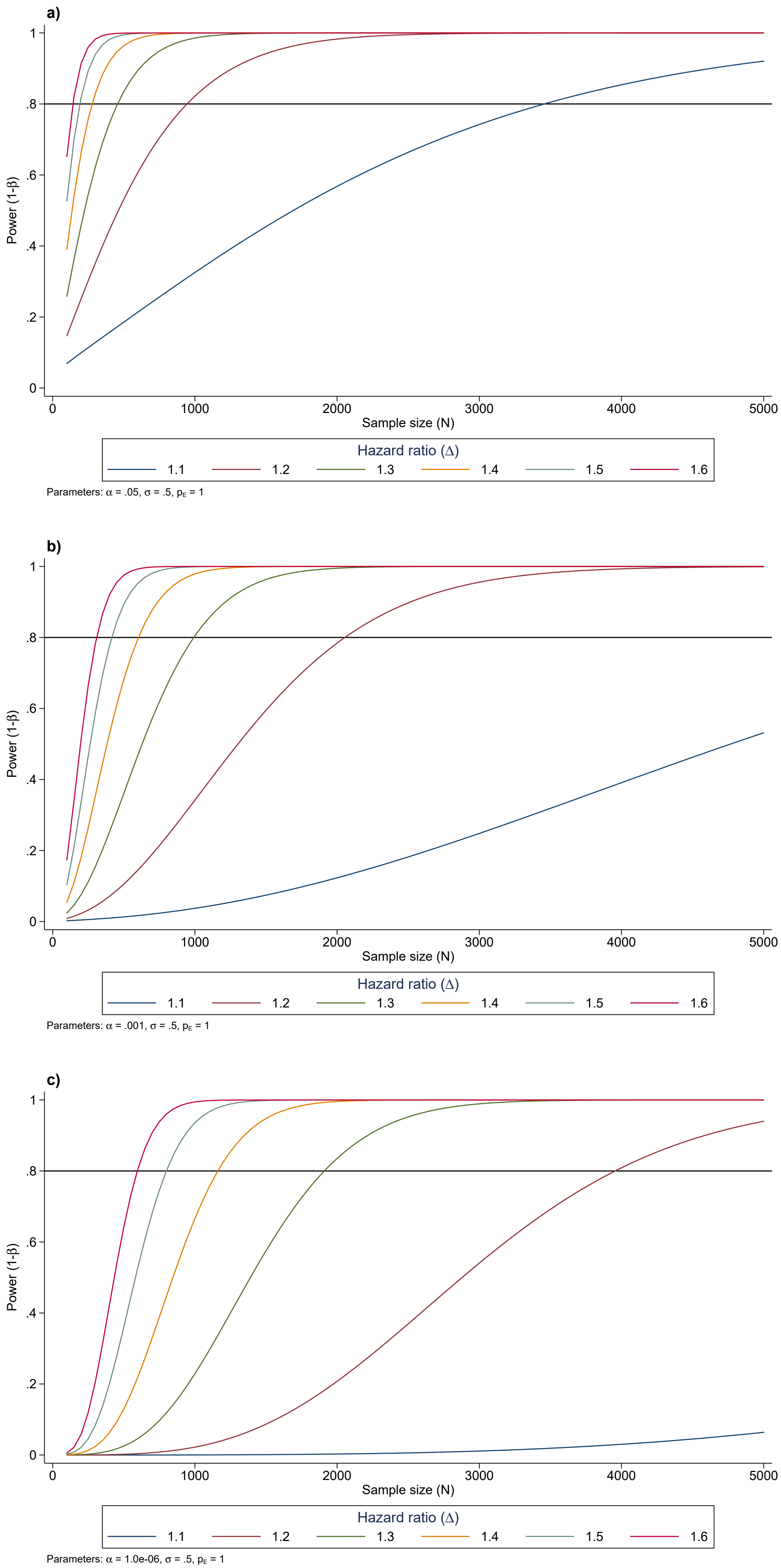

Supplementary Figure 1: Statistical power estimations for diet and cancer associations for α levels of 0.05 (panel a),0.001 (panel b) and 0.000001 (panel c) by number of cancer cases and hazard ratios
