# Supplementary material for: An umbrella review of the evidence associating diet and cancer risk at 11 anatomical sites
Source: Nat Commun. 2021 Jul 28;12:4579. doi: 10.1038/s41467-021-24861-8 (PMC8319326; doi:10.1038/s41467-021-24861-8)
Supplement: Supplementary file 3 — Description of Additional Supplementary Files [file 41467_2021_24861_MOESM3_ESM.pdf]

Description of Additional Supplementary Files

Supplementary Data 1: General characteristics, grading of the evidence, estimated number of future studies required to achieve at least 80% conditional power, and Rosenberg's fail-safe number of the 860 meta-analyses investigating the associations of diet and cancer risk at 11 anatomical sites.

Supplementary Data 2: Evaluation of heterogeneity, small study effects end excess significance bias in the 860 meta-analyses investigating the associations of diet and cancer risk at 11 anatomical sites

Supplementary Data 3: Descriptive statistics of the meta-analyses by exposure category included in the umbrella review grading the evidence on diet and cancer risk.

Supplementary Data 4: Number and percentage of meta-analyses by exposure category which meet the individual and the overall criteria used for the grading of the evidence on diet and cancer risk.

Supplementary Data 5: General characteristics of the 27 not statistically significant meta-analyses investigating the associations of diet and cancer risk, where the estimated number of additional studies that could provide sufficient power to drive summary estimates to nominal significance was less than the number of studies included in the current meta-analyses, suggesting further study could change inferences.

Supplementary Data 6: List of individual studies included in the current umbrella review.

Supplementary Data 7: Code for umbrella review on binary outcomes.
